# Health needs of internally displaced persons from the Catatumbo region, Colombia: 2025

**DOI:** 10.21203/rs.3.rs-10580751/v1

**Published:** 2026-09-04

**Authors:** Lianet Vazquez, Angela Restrepo, Lendy Rojas Bautista, Gustavo Rangel, Denisse Pareja Valarezo, Lindsey Martin, Zahra Shah, Ramzy Alaeddine, Andrés F. Henao Martínez, Camilo Avila Paerez, Mahmoud Kannas, Carlos Franco Paredes, Amir M. Mohareb

**Affiliations:** Brigham and Women’s Hospital; MedGlobal Latin America; MedGlobal Latin America; MedGlobal Latin America; MedGlobal Latin America; Massachusetts General Hospital; MedGlobal Latin America; MedGlobal Latin America; University of Colorado Denver; MedGlobal Latin America; MedGlobal Latin America; Hospital Infantil de México Federico Gómez. México City; Massachusetts General Hospital

**Keywords:** Internally Displaced Persons, Respiratory infections, Humanitarian medicine, Refugee, Non-Communicable Diseases, Mental Health

## Abstract

**Background:**

Violence erupted in the Catatumbo region of Colombia in January 2025, leading to internal displacement of Colombians and double displacement of Venezuelan persons residing in the region as immigrants, holding temporary protected status. We estimated the disease prevalence and health needs among the displaced.

**Methods:**

We conducted a cross-sectional study of displaced persons from Catatumbo receiving medical care at 29 clinical sites in Cúcuta city in Norte de Santander, Colombia, from January to May 2025. We categorized diagnoses and symptoms as infectious or non-infectious, with organ-system subgroups according to International Classification of Diseases – 10th Revision (ICD-10) classification. We calculated the prevalence of health conditions by dividing the number of diagnoses by the total number of patients. We used multivariable logistic regression to estimate adjusted odds ratios (aORs) with 95% confidence intervals (CIs) for associations between sociodemographic factors and health conditions.

**Results:**

There were 2002 internally displaced persons (median age 25 years, 59.4% women) evaluated in medical encounters: 1454 (72.6%) were Colombian, and 477 (23.8%) were originally from Venezuela. The most prevalent diagnoses were infectious diseases (1401, 70%), with high burden of respiratory (681, 34%), gastrointestinal (310, 15.5%), genitourinary (302, 15.1%), and skin infections (73, 3.6%). Less prevalent diagnoses included non-communicable diseases of cardiometabolic and mental health origin (100, 5%), musculoskeletal and connective tissue disorders (n = 95, 4.7%), and dermatologic conditions (n = 77, 3.8%). Children were more likely to have respiratory infection (aOR 2.62, 95% CI: 2.09–3.29) and gastrointestinal diseases (aOR 1.62, 95% CI: 1.21–2.18), while adults had a higher prevalence of non-communicable diseases (aOR 3.35, 95% CI: 2.08–5.42) and musculoskeletal disorders (aOR 2.56, 95% CI: 1.57–4.18). Genitourinary infections were more common in people of Venezuelan origin (aOR 1.45, 95% CI: 1.08–1.94) and least common in men (aOR 0.33, 95% CI: 0.23–0.45).

**Conclusions:**

Internally displaced persons from Catatumbo, Colombia, including doubly displaced Venezuelans, had wide-ranging health needs. Developing a comprehensive emergency response approach that includes screening, acute management, and referral pathways for chronic conditions is imperative to address acute needs and reduce long-term morbidity and mortality.

## Background

Forced displacement has grown over the past decade and poses major challenges to health and healthcare delivery at national and global levels. As of June 2025, there were 117.5 million forcibly displaced persons worldwide, a designation defined by the UN High Commissioner for Refugees (UNHCR) to include 42.5 million refugees, 67.8 million internally displaced persons (IDPs), and 8.42 million asylum seekers.^[1]^ Refugees and asylum seekers flee their home country with a credible fear of persecution, whereas IDPs face displacement within national boundaries.^[2,3]^ While refugees and asylum seekers are afforded international protections,^[4]^ internally displaced persons often lack similar safeguards or access to international humanitarian assistance.^[5,6]^ Forced displacement may result in fragmentation of health services, contributing to gaps in preventative and longitudinal medical care, which in turn leads to increased morbidity and mortality.^[7,8]^

Forced displacement in Colombia is unique. Of the 21.9 million forcibly displaced persons across the Americas in 2025, Colombia is home to an estimated 7 million internally displaced persons – the largest in the region and third largest in the world – in addition to nearly 3 million displaced persons from Venezuela.^[1,9]^ The Norte de Santander department in Colombia has the highest per capita concentration of displaced persons from Venezuela, ranging from 17–23% of its population, while it has also seen an increase in violence contributing to ongoing displacement.^[10]^ Displaced Venezuelans are granted temporary protection status under Colombian law.^[11]^ In January 2025, renewed armed conflict among non-state actors erupted in the Catatumbo region, in Norte de Santander, which displaced more than 110,000 people (Fig. 1).^[12,13]^ This crisis led to a high number of Colombian internally displaced persons in a short period of time and a high number of “doubly displaced” families of Venezuelan origin. Double displacement refers to situations in which a group of people who were displaced once before, become forcibly removed a second time.^[14]^

While research on refugee and migration health in Latin America has grown, less has been published on the health of internally displaced persons.^[15–19]^ To our knowledge, no current study exists quantifying the acute health needs of refugees and IDPs resulting from the recent violent escalation in Catatumbo. Moreover, there remain unanswered questions about the unique health needs of those who experience double displacement. The objective of this study is to quantify disease prevalence among internally displaced and doubly displaced communities in Catatumbo, Colombia, in the aftermath of the most recent escalation of violence.

## Methods

### Study design

We conducted a cross-sectional study of primary data collected at 29 clinical sites in Cúcuta, Colombia, from January 25, 2025, to May 22, 2025. The clinical sites were operated by MedGlobal, an international humanitarian relief organization that has been working in Colombia since 2019. Patients at these clinical sites were exclusively families displaced from the Catatumbo region. We extracted electronic health data from the medical relief clinics, including demographic data, reported symptoms, and diagnoses. We analyzed the most common medical conditions diagnosed for this population and demographic factors associated with them. This study received approval from the Massachusetts General Hospital’s institutional review board (IRB) and approval from the MedGlobal leadership.

### Setting and Population

Of the more than 110,000 persons displaced during the Catatumbo crisis, 2010 received medical care at MedGlobal clinics during the study period. Clinical sites included shelters, hotels, stadiums, and community centers dispersed throughout the city of Cúcuta, with a focus on urban areas as part of a coordinated emergency response. Patient outreach was conducted primarily through local community organizations, which disseminated the dates and times for clinical visits. Some clinics were located at the site where patients were lodging (i.e., hotels and shelters), whereas others were situated in more public areas (i.e., stadiums and community centers), which were most easily accessible to IDPs. The clinic provided access to healthcare services without discrimination based on ethnicity, race, insurance, or socioeconomic status. All medical care provided was free to the patient. Diagnoses were made based on clinical history and physical exam, with limited access to point-of-care laboratory testing or ultrasound. When appropriate, patients received medications and referrals to receive ongoing care through coordination with governmental and non-governmental organizations operating in the region. Patients with mental health needs as primary complaint were frequently referred to partner organizations specifically equipped to provide mental and behavioral health and were not otherwise evaluated by the MedGlobal clinical team.

### Data collection

We collected primary electronic medical data, including demographics (age, sex, nationality), month of visit, insurance type, clinical site, patient-reported symptoms, and diagnoses made during the visit. Symptoms and diagnoses were reported as free text in Spanish, with ample variation within clinical entries. A qualified bilingual – English and Spanish – medical doctor translated the symptoms and diagnoses included in the raw data into English. Some encounters recorded multiple diagnoses per patient, without distinction between primary and secondary diagnoses. When this happened, we counted each diagnosis as a unique observation. Other encounters only recorded patient-reported symptoms, without a clinical diagnosis. We excluded data entries without either a diagnosis or a symptom documented from the final analysis.

### Covariates

We classified nationality as Colombian, Venezuelan, or unspecified if none was provided. We grouped service locations into the following categories: hotel, shelter, community center, stadium, or other. Insurance types included Comfaoriente, Coosalud, Nueva EPS, other, or none, which we encoded as binary for the analysis.

### Outcomes

We categorized symptoms and diagnoses following the ICD-10 classification. We grouped diagnoses into infectious and non-infectious categories, with subcategorization by organ system and non-communicable disease (NCD) classification. We defined NCDs to include mental health diagnoses and the four major types of non-infectious conditions contributing to the highest morbidity and mortality: cardiovascular diseases, diabetes, chronic respiratory diseases, and cancers.^[20,21]^ The infectious categories included respiratory, gastrointestinal, genitourinary, and dermatologic infections. Due to lack of confirmatory testing, infectious diagnoses were made clinically or based on presumptive treatment. We assumed diagnoses of gastritis to be secondary to *H. pylori* infection, and tabulated parasitic infections based on empiric treatment. The non-infectious categories included non-communicable diseases (with subcategories of hypertension, hyperlipidemia, diabetes mellitus, cancer, asthma, and mental health), musculoskeletal and connective tissue disorders, dermatologic, and other (with subcategories for pregnancy, anemia, venous insufficiency, neurologic disorders, and physical trauma). We grouped symptoms into general, infectious, and pain categories.

### Statistical analysis

We calculated descriptive statistics for all clinical encounters and stratified them by patient nationality (Colombian, Venezuelan, or Unspecified), age group (< 18, 18–25, 26–49, 50–64, > 65 years), insurance status (insured versus uninsured), and service location (shelter, hotel, stadium, community center, or other). We computed the prevalence of diagnosis categories by dividing the number of diagnoses by the total number of patients. Because one patient can have multiple conditions at once, the sum of the diagnosis categories exceeds the total number of patients with diagnoses. We separately calculated the prevalence of symptoms, focusing on non-specific infectious symptoms (e.g., undifferentiated fever) and non-specific pain syndromes. Where only patient-reported symptoms were recorded, we did not infer or assign a diagnosis. We stratified prevalence by nationality and age group. We used multivariable logistic regression to characterize the factors associated with each diagnostic category, reporting odds ratios with 95% confidence intervals (CIs). Because access to healthcare services as part of the humanitarian response in Catatumbo was extended to all persons affected independent of insurance status, we analyzed two separate models, one including insurance status as a potential factor, and another excluding it. We used R version 4.5.2 for the analysis using *forestplot* and *tmap* packages.

## Results

The study sample included 2010 unique participants, of whom 2002 (99.6%) had documented symptoms and/or diagnoses and were included in the final analysis (Table 1). The patients included in the study represented less than 2% of the total reported displaced population from the Catatumbo crisis. The sample was mostly women (1190, 59.4%) with a median (IQR) age of 25 (10–41) years. There were two main nationalities represented – Colombian and Venezuelan – with 1454 (72.6%) from Colombia. Most people had health insurance (1561, 78%), although for people of Venezuelan origin, most were uninsured (289, 60.6%). The clinical sites with the highest participant volume were shelters (870, 43.5%), followed by hotels (529, 26.4%) and stadiums (441, 22%). A total of 499 (24.9%) participants had only a symptom recorded, with no diagnosis.

Infectious diseases had the highest prevalence (1401, 70%), compared to non-infectious diseases (358, 17.9%). Among infectious diseases, respiratory illness had the highest burden (681, 34%), with upper respiratory tract infections accounting for most cases (396, 19.8%). Gastrointestinal infections were present in 310 (15.5%) clinical evaluations, with encounters for presumptive or symptom-based intestinal parasitic treatment accounting for 254 cases (12.7%), followed by gastritis (56, 2.8%), presumed to be due to *H. pylori* infection. Genitourinary infections were present in 302 (15.1%) evaluations, including urinary tract infections (244, 12.2%) and vaginosis (58, 2.9%). Dermatologic infections (73, 3.6%) included mycoses (36, 1.8%), cellulitis (23, 1.1%), and skin abscess (11, 0.5%).

Among non-infectious diagnoses, NCDs had the highest prevalence (100, 5%), followed by musculoskeletal and connective tissue disease (95, 4.7%), and dermatologic conditions (77, 3.8%). The NCD diagnoses included hypertension (55, 2.7%), asthma (14, 0.7%), hyperlipidemia (11, 0.5%), mental health disorder (9, 0.4%), and diabetes mellitus (7, 0.3%). Musculoskeletal and connective tissue diagnoses included contracture (n = 49, 2.4%), non-inflammatory arthritis (n = 19, 0.9%), inflammatory arthritis (n = 15, 0.7%), and an all-encompassing other category (n = 12, 0.6%) that aggregated less prevalent diagnoses such as bursitis and fractures. Dermatitis (69, 3.4%) was most prevalent among non-infectious dermatologic diagnoses. Other dermatologic conditions (8, 0.4%) included unspecified rash, lipoma, burns, unspecified bite, and purpuric rash. Non-infectious diagnoses with lower prevalence included anemia (20, 1%), neurologic conditions such as epilepsy and stroke (20, 1%), pregnancy (n = 9, 0.4%), and injuries resulting from trauma (5, 0.2%).

Participant-reported symptoms followed a similar distribution. Infectious symptoms were most prevalent (717, 35.8%), with cough (372, 18.6%), unspecified viral symptoms (191, 9.5%), diarrhea (86, 4.3%), and fever (27, 1.3%) as the most widely reported. Pain symptoms were the second most widely reported (383, 19.1%), including headache (114, 5.7%), myalgias (58, 2.9%), and lower back pain (49, 2.4%).

Children had higher prevalence of respiratory infections (aOR 2.62, 95% CI 2.09–3.29) and gastrointestinal diseases (aOR 1.62, 95% CI 1.21–2.18), whereas adults had higher prevalence of non-communicable diseases (aOR 3.35, 95% CI 2.08–5.42), and musculoskeletal and connective tissue disorders (aOR 2.56, 95% CI 1.57–4.18) (Fig. 2). People of Venezuelan origin had a higher prevalence of genitourinary infections (aOR 1.45, 95% CI 1.08–1.94). Gastrointestinal diseases were most prevalent in male patients (aOR 1.38, 95% CI 1.07–1.77).

Individuals evaluated at community centers had a higher prevalence of gastrointestinal illness (aOR 2.49, 95% CI 1.63–3.79) but a lower prevalence of respiratory infections (aOR 0.56, 95% CI 0.36–0.84) compared to those evaluated at hotels. Respiratory infections were also less prevalent in people evaluated at stadiums (aOR 0.75, 95% CI 0.57–0.99). The prevalence of reported pain symptoms was higher among patients of unspecified nationality (aOR 2.46, 95% CI 1.44–4.14), while it was lower in pediatric patients (aOR 0.21, 95% CI 0.15–0.30). People evaluated in shelters (aOR 2.09, 95% CI 1.48–3.00), community centers (aOR 2.65, 95% CI 1.62–4.30), or stadiums (aOR 1.73, 95% CI 1.17–2.59) had a higher prevalence of reported pain symptoms compared to those evaluated in hotels. Insurance status was not associated with higher or lower prevalence of any medical condition or symptom.

## Discussion

This cross-sectional study examines disease prevalence among internally displaced and doubly displaced persons from Catatumbo, in the Norte de Santander department of Colombia. Our results can be summarized into three main findings. First, there was a high prevalence of infections (70%), with highest burden of respiratory infections (34%), followed by gastrointestinal illness (15.5%), genitourinary (15.1%) and dermatologic infections (3.6%). Second, we found a surprisingly low prevalence of mental health diagnoses (0.4%) with overall low burden of NCDs (5%). Third, with few exceptions, internally displaced Colombians had similar disease prevalence as doubly displaced Venezuelans. Notably insurance status also did not lead to different prevalence rates. The findings from our study represent, to our knowledge, the most comprehensive evaluation of the health needs of internally displaced persons from Catatumbo, with implications for humanitarian emergency response preparedness.

Despite the high reported prevalence of infections in our study (70%), it is possible that this figure underestimates true burden. We found children and people living in congregate spaces such as shelters and hotels to be most affected. Reynolds et al. ^[22]^ reported a similar distribution of illness in asylum seekers at a settlement camp at the US-Mexico border, citing respiratory infections as the most frequent diagnosis, with the highest burden in children. A 2006 UNHCR report also cited a high burden of infections among the top 10 causes of morbidity, affecting IDPs in Colombia at a higher rate compared to the host population.^[16]^ The risk of infections may increase with displacement through limited access to clean water, sanitation, hygiene services, overcrowding, and possible biological pathways such as elevated cortisol levels contributing to decreased immunity.^[23]^ In our study, symptoms such as cough (18.6%), unspecified viral symptoms (9.5%), headache (5.7%), diarrhea (4.3%), myalgias (2.9%), and fever (1.3%) were also common. Given that, for some participants, we had only reported symptoms without diagnoses, the total prevalence of infectious diseases was likely higher than 70% if we attribute at least some symptoms to undiagnosed infections. Diagnoses not explicitly captured likely included dengue, which can present with headache, fever, and myalgias. Indeed, Colombian public health officials reported at least 232 cases of dengue in Norte de Santander during the January to June 2025 period.^[24]^ In addition to integrating sanitation and hygiene services into emergency response during displacement crises, it would be important to increase diagnostic capacity through serologic confirmatory testing to identify key pathogenic targets that can guide immunization campaigns and other preventive interventions for displaced populations.

Despite the recorded violence in Catatumbo^[12]^ and the well-documented burden of mental health diagnoses in the region,^[17]^ few patients reported physical trauma or mental health complaints in our study. We found pain syndromes, which have previously been linked to depression in a bidirectional relationship^[25,26]^ - had a 19% prevalence, but these included headaches and myalgias, which may overlap with infectious diagnoses. Several reasons could have contributed to the low relative prevalence of mental health and psychiatric diagnoses. First, the triaged patients suffering from primary mental health complaints may have received care through partnering organizations with specialty behavioral health counseling as part of the emergency response. Those records were not available for this analysis. Second, mental health conditions are likely under-reported in this context due to hesitancy to disclose, as has been noted in other similar studies.^[22]^ It is possible that patients under-reported, physicians under-recorded, or under-diagnosed mental health complaints due to the sensitive nature of the security situation. Lastly, direct trauma may not have impacted the specific population of displaced persons evaluated in this study as much as it did those who remained in the region most affected. The World Health Organization (WHO) has recognized a significant treatment gap in humanitarian settings where exposure to psychological trauma creates a high mental health burden, which far exceeds available services.^[27]^ Ongoing investment in prompt detection and treatment of mental health disturbances in humanitarian crises remains an essential priority area.

Non-communicable diseases tend to produce long-term exacerbations in the medium term (months to years) following displacement events due to interruptions in primary care and medication access.^[28–30]^ Our study showed total NCD prevalence of 5%, with hypertension accounting for the highest burden (2.7%) and diabetes for the lowest burden (0.3%). These results are significantly lower compared to the national average of 22% prevalence for hypertension, followed by diabetes at 5.7%, and likely do not represent true NCD prevalence in the displaced population.^[31]^ The discrepancy is attributed in part to the relatively young population included in our sample. Our study also occurred as part of services embedded within the emergency response to a humanitarian crisis, which traditionally prioritize addressing acute needs such as infectious diseases and traumatic injuries, rather than NCDs.^[32]^ With limited testing capabilities, there was decreased diagnostic capacity to screen for new cases or ascertain whether acute presentations represented exacerbations of undiagnosed chronic conditions. Our analysis also took place in the immediate term after the beginning of a new displacement crisis, which may have been insufficient time to evaluate the medium to long-term impact of NCDs in that population. The WHO has recognized the increasing burden of morbidity and mortality attributed to NCDs,^[20]^ prompting renewed efforts to integrate NCD-directed care into the humanitarian response. ^[32,33]^ Integrating screening, treatment, and referral pathways for management of NCDs among displaced persons is imperative to provide continuity of care, prevent exacerbations, and overall mortality.

Whereas the prevalence of most diseases was not affected by nationality or insurance status, genitourinary diseases were 41% more prevalent among doubly displaced people of Venezuelan origin, compared to internally displaced Colombians. The difference in genitourinary disease burden may be related to the higher overall prevalence of sexually transmitted infections among the Venezuelan population due to increased vulnerability to sexual exploitation and irregular migration status without reliable access to health insurance for prompt screening and treatment.^[34]^ Two separate studies found the prevalence of syphilis and Human Immunodeficiency Virus (HIV) in Venezuelans in Colombia to be 5.1% and 0.9%, respectively, compared to 1.3–1.4% for syphilis and 0.5% for HIV among the non-displaced Colombian and Venezuelan adult population.^[35,36]^ The otherwise apparent parity in outcomes between the two groups across other diagnosis categories and insurance status highlights the possibility of a shared vulnerability to the disruption to healthcare access intrinsic to forced displacement.^[7]^ Insurance registration requirements that IDPs from the municipality of origin must complete in the new municipality of residence following displacement,^[37]^ can disrupt continuity of coverage for those newly displaced, as well as for those facing double displacement who may have lacked insurance coverage to begin with. Our study may also have been underpowered to detect true differences between the two populations, and therefore not adequately capture the health impacts of double displacement. This persistent lacuna has important implications for future studies to better understand acute versus compounded vulnerability in double displacement to guide interventions and resource allocation. Ensuring ongoing equitable access to healthcare services following humanitarian crises is pivotal, and it includes provision of direct medical services as well as rapid integration of displaced persons into existing healthcare delivery systems.

Our study had several limitations. The cross-sectional study design precludes causal inference. The associations we found may be subject to confounding by unmeasured variables. Our analysis relied on clinical documentation conducted with limited resource availability and in settings lacking confirmatory diagnostic testing, which limits the specificity of diagnoses. It is possible that the same medical conditions were recorded differently between clinicians. Due to the nature of the study design, we only captured conditions that presented for medical attention, which represented less than 2% of the total displaced population affected by the crisis. Prior studies have shown that facility-based data such as the one collected in this study tends to underestimate disease burden compared to population-based surveys.^[38,39]^ The study population was also primarily women and relatively young, which limits the generalizability of the findings.

## Conclusions

In conclusion, this study contributes valuable findings to better understand the health needs of displaced persons from Catatumbo, Colombia, and inform future health-related interventions targeting internally displaced persons. The results highlight the need to screen and identify mental health disorders as a key best practice initiative given the likely under-evaluated and/or under-reported nature of these conditions among the internally displaced population. For the containment and treatment of highly prevalent infectious diseases, congregate settings designated for sheltering displaced persons would ideally be equipped with basic hygiene kits, access to potable water, mosquito nets, and quarantine capabilities in the event of outbreaks. Incorporating protocols for NCD screening and management could also improve detection and decrease exacerbations and morbidity. These efforts would require integrating internally displaced persons into existing healthcare systems to ensure continuity of care and coordinating with regional and local actors, including public health authorities and non-profit organizations. Health is an important determinant of wellbeing and individual and social development. We conclude that addressing health needs amongst the most vulnerable populations including internally displaced persons must be considered within a framework of human rights and social justice.^[40]^

## Figures and Tables

**Figure 1:**
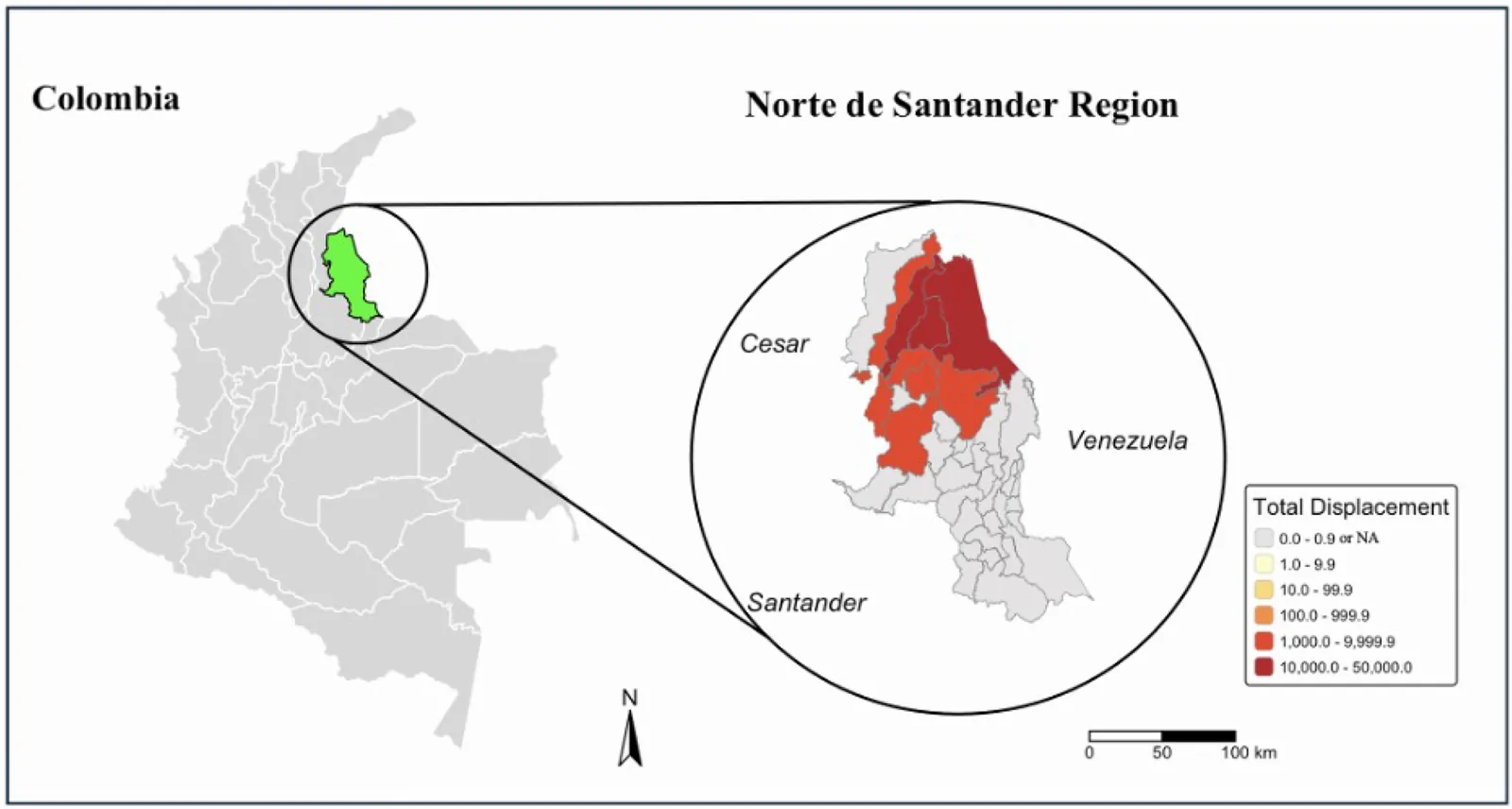
Displacement in the Catatumbo area within Norte de Santander, Colombia, from January to May 2025. Catatumbo is in the Norte de Santander department of Colombia. It is comprised of 11 municipalities including Tibú, El Tarra, Teorama, Convención, San Calixto, Hacarí, La Playa, Sardinata, El Carmen, Ábrego, and Ocaña. Using national data,^[41]^ we produced a heat map in logarithmic scale of displacement data for Catatumbo municipalities from January 2025 to May 2025. A total of 88,037 persons were displaced during this period, with highest displacement in Tibú (43,000), Teorama (14000) and El Tarra (13000).

**Figure 2:**
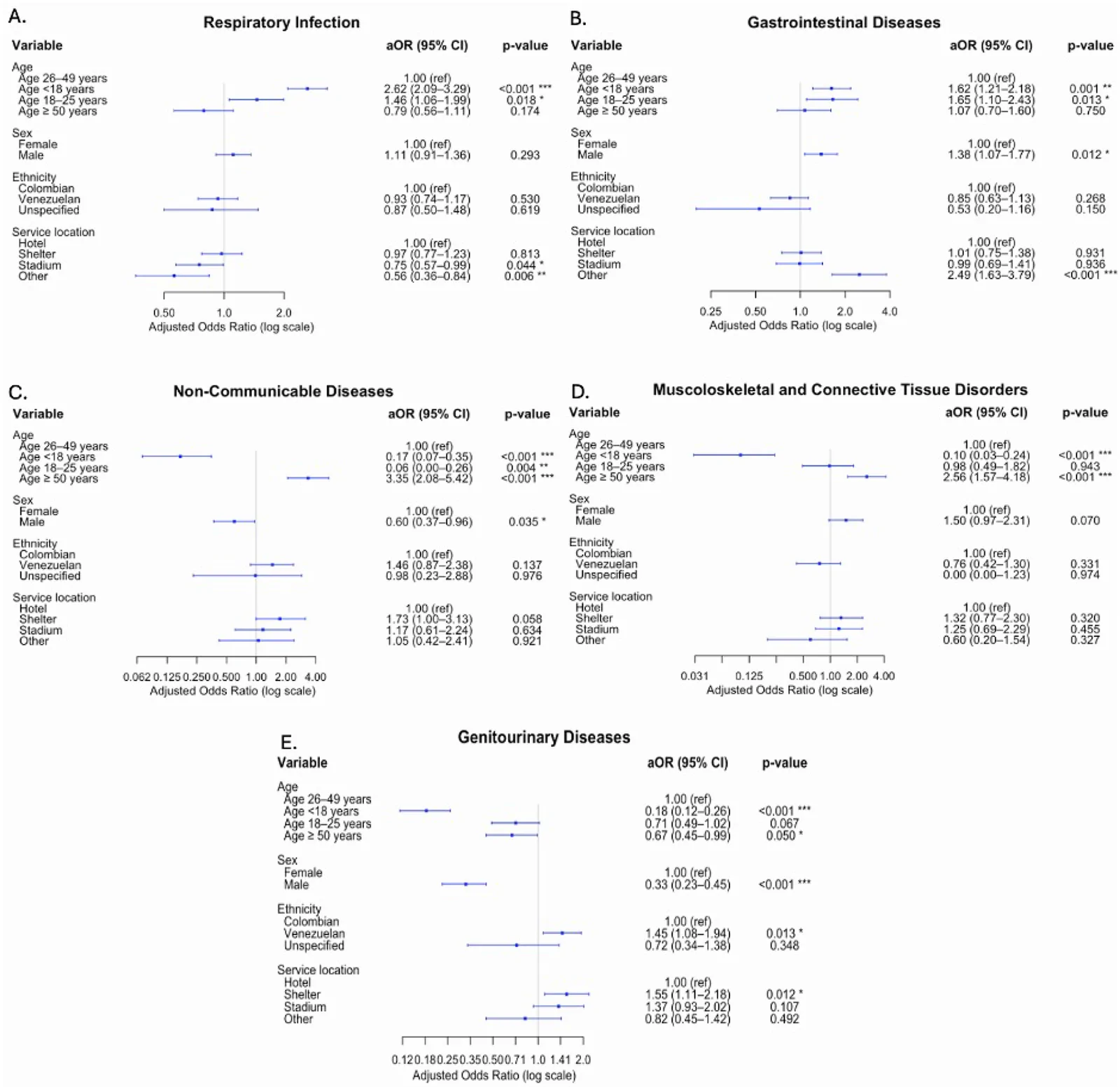
Demographic factors associated with the top diagnosis categories in Cúcuta, from January to May 2025. We built forest plots to characterize the demographic factors associated with the top 5 most prevalent diagnosis categories among patients evaluated at MedGlobal clinical sites in Cúcuta, Colombia. Panel A represents all respiratory infections. Panel B represents all gastrointestinal conditions, including infectious and non-infectious diagnoses. Panel C represents non-communicable diseases, defined in our study to include hypertension, hyperlipidemia, diabetes, mental health diagnoses, cancer and chronic respiratory conditions. Panel D represents musculoskeletal and connective tissue disorders. Panel E represents genitourinary diseases, including vaginosis and urinary tract infections.

**Table 1:** Demographics of patients evaluated at clinical sites in Cúcuta, Colombia, from January to May 2025. *(To be included in the* results *section of the manuscript)*

Demographics		All	Colombian	Venezuelan	Unspecified
		n (%)	n (%)	n (%)	n (%)
		2002 (100.0%)	1454 (72.6%)	477 (23.8%)	71 (3.5%)
**Gender**					
	Female	1190 (59.4%)	820 (56.4%)	320 (67.1%)	50 (70.4%)
	Male	812 (40.6%)	634 (43.6%)	157 (32.9%)	21 (29.6%)
**Age, years**					
	Median [IQR]	25 [10–41]	26 [8–45]	23 [12–36]	28 [20–34]
**Age group, years**					
	< 18	760 (38.0%)	552 (38.0%)	194 (40.7%)	14 (19.7%)
	18–25	256 (12.8%)	169 (11.6%)	70 (14.7%)	17 (23.9%)
	26–49	711 (35.5%)	496 (34.1%)	177 (37.1%)	38 (53.5%)
	50–64	196 (9.8%)	165 (11.3%)	29 (6.1%)	2 (2.8%)
	> 65	79 (3.9%)	72 (5.0%)	7 (1.5%)	0 (0.0%)
**Insurance Status**					
	Insured	1561 (78.0%)	1357 (93.3%)	183 (38.4%)	21 (29.6%)
	Uninsured	416 (20.8%)	77 (5.3%)	289 (60.6%)	50 (70.4%)
	Unknown	25 (1.2%)	20 (1.4%)	5 (1.0%)	0 (0.0%)
**Service Location**					
	Shelter	870 (43.5%)	605 (41.6%)	208 (43.6%)	57 (80.3%)
	Hotel	529 (26.4%)	380 (26.1%)	149 (31.2%)	0 (0.0%)
	Stadium	441 (22.0%)	352 (24.2%)	75 (15.7%)	14 (19.7%)
	Community Center	154 (7.7%)	110 (7.6%)	44 (9.2%)	0 (0.0%)
	Other	8 (0.4%)	7 (0.5%)	1 (0.2%)	0 (0.0%)

Of 2010 patients evaluated, 2002 had documented symptoms, diagnoses, or both, and were included in the final analysis. We calculated descriptive statistics for demographic factors and stratified by nationality. IQR stands for interquartile range.

**Table 2:** Disease prevalence by infectious versus non-infectious categories, in Cúcuta, Colombia, from January to May 2025. *(To be included in the* results *section of the manuscript)*

Category	Diagnosis	Prevalence [N = 2002 patients]
		n (%)
**Infectious**		1401 (70%)
Respiratory		681 (34%)
	Upper Respiratory Tract Infection	396 (19.8%)
	Unspecified Respiratory Tract Infection	280 (14%)
	Lower Respiratory Tract Infection	5 (0.2%)
Gastrointestinal		310 (15.5%)
	Presumptive or treatment-based parasitic infections	254 (12.7%)
	Gastritis	56 (2.8%)
Genitourinary		302 (15.1%)
	Urinary Tract Infection	244 (12.2%)
	Vaginosis	58 (2.9%)
Dermatologic		73 (3.6%)
	Mycoses	36 (1.8%)
	Cellulitis	23 (1.1%)
	Abscess	11 (0.5%)
	Other	3 (0.1%)
**Non-Infectious**		358 (17.9%)
Non-Communicable Diseases		100 (5%)
	Hypertension	55 (2.7%)
	Asthma	14 (0.7%)
	Hyperlipidemia	11 (0.5%)
	Mental Health Disorder	9 (0.4%)
	Diabetes Mellitus	7 (0.3%)
Musculoskeletal and Connective Tissue Disorders		95 (4.7%)
	Contracture	49 (2.4%)
	Non-inflammatory arthritis	19 (0.9%)
	Inflammatory arthritis	15 (0.7%)
	Other	12 (0.6%)
Dermatologic		77 (3.8%)
	Dermatitis	69 (3.4%)
	Other	8 (0.4%)
Other		
	Anemia	20 (1%)
	Neurological	20 (1%)
	Pregnancy	9 (0.4%)
	Venous insufficiency	7 (0.3%)
	Trauma	5 (0.2%)
	Other	8 (0.4%)
**No Diagnosis Recorded**		499 (24.9%)

Disease prevalence was calculated by tallying the total number of unique diagnosis occurrence in patients evaluated at 29 clinical sites in Cúcuta city, Colombia, and dividing it by the total number of patients. Of 2002 patients, 499 did not have a diagnosis recorded. The remainder had one or multiple diagnoses recorded per clinical encounter, without a distinction between primary and secondary.

## Data Availability

The data that support the findings of this study are available from MedGlobal but restrictions apply to the availability of these data, which were used under license for the current study, and so are not publicly available. Data are however available from the authors upon reasonable request and with permission of MedGlobal.

## List of abbreviations

aORs: Adjusted odds ratios
CI: Confidence intervals
HIV: Human Immunodeficiency Virus
ICD-10: International Classification of Diseases – 10^th^ Revision
IDPs: Internally displaced persons
IQR: Interquartile range
IRB: Institutional Review Board
NCD: Non-communicable disease
UNHCR: UN High Commissioner for Refugees
WHO: World Health Organization
